# Do Insect Populations Die at Constant Rates as They Become Older? Contrasting Demographic Failure Kinetics with Respect to Temperature According to the Weibull Model

**DOI:** 10.1371/journal.pone.0127328

**Published:** 2015-08-28

**Authors:** Petros Damos, Polyxeni Soulopoulou

**Affiliations:** 1 Department of Crop Production, Faculty of Agriculture, Forestry and Natural Environment, Aristotle University of Thessaloniki, Thessaloniki, Greece; 2 Department of Environment, University of the Aegean, Mytilene, Lesvos, Greece; Université de Perpignan Via Domitia, FRANCE

## Abstract

Temperature implies contrasting biological causes of demographic aging in poikilotherms. In this work, we used the reliability theory to describe the consistency of mortality with age in moth populations and to show that differentiation in hazard rates is related to extrinsic environmental causes such as temperature. Moreover, experiments that manipulate extrinsic mortality were used to distinguish temperature-related death rates and the pertinence of the Weibull aging model. The Newton-Raphson optimization method was applied to calculate parameters for small samples of ages at death by estimating the maximum likelihoods surfaces using scored gradient vectors and the Hessian matrix. The study reveals for the first time that the Weibull function is able to describe contrasting biological causes of demographic aging for moth populations maintained at different temperature regimes. We demonstrate that at favourable conditions the insect death rate accelerates as age advances, in contrast to the extreme temperatures in which each individual drifts toward death in a linear fashion and has a constant chance of passing away. Moreover, slope of hazard rates shifts towards a constant initial rate which is a pattern demonstrated by systems which are not wearing out (e.g. non-aging) since the failure, or death, is a random event independent of time. This finding may appear surprising, because, traditionally, it was mostly thought as rule that in aging population force of mortality increases exponentially until all individuals have died. Moreover, in relation to other studies, we have not observed any typical decelerating aging patterns at late life (mortality leveling-off), but rather, accelerated hazard rates at optimum temperatures and a stabilized increase at the extremes.In most cases, the increase in aging-related mortality was simulated reasonably well according to the Weibull survivorship model that is applied. Moreover, semi log- probability hazard rate model illustrations and maximum likelihoods may be usefully in defining periods of mortality leveling off and provide clear evidence that environmental variability may affect parameter estimates and insect population failure rate. From a reliability theory standpoint, failure rates vary according to a linear function of age at the extremes indicating that the life system (i.e., population) is able to eliminate earlier failure and/or to keep later failure rates constant. The applied model was able to identify the major correlates of extended longevity and to suggest new ideas for using demographic concepts in both basic and applied population biology and aging.

## Introduction

The force of mortality is an important determinant of the population dynamics of a species, and an understanding of mortality forces contributes in the mathematical description of aging in living systems and the development of better management strategies for insect pests. Although all biological systems, including insects, are subject to aging, the fundamental mechanism underling the force of mortality are far from clear[1]. The force of mortality refers to the instantaneous death rate at a certain age measured for each individual. It is identical in concept to failure rate, also called hazard function, in reliability theory. Nevertheless, since the structure of biological systems (i.e. Insect population) changes in time and space due to outside components (i.e. Abiotic factors), their mortality may differ under variable conditions.

In poikilotherms and insects in particular, there are significant concerns that should be brought into account when modeling the force of mortality [2]. Insect life span and aging, for instance, are strongly affected by climatic conditions in which temperature dominates while other examples include changes in biochemical rhythms as affected by photoperiod [3]. Nevertheless, most aging models are used only in populations that were kept at reference temperatures (usually the optimum) and have not examined death rates at the extremes [4, 5]. Moreover, well-established hazard models are used on insect-model species that deliver some positive benchmarks; these model species include Diptera, and *Drosophila melanogaster* and the Mediterranean fruit fly *Ceratitis capitata* [6, 7] and are more often used because they are well-maintained in laboratory conditions and they have high reproductive potential and longevity. On the other hand, short-lived species such as the moth Lepidoptera have received virtually no attention, and temperature has rarely been taken into account when fitting survivorship models. Thus, it is actually unknown whether a traditional model (e.g., The Weibull or Gompertz) is capable to fit survivorship curves under different temperature scenarios and at advanced ages, which is where other models go wrong.

From an insect’s demography standpoint, age and stage-structured laboratory population studies contribute importantly to the illumination of pest population dynamics. Regional and in-seasonal periodic oscillations could be explained for instance by observing population stability and structure after analyzing major life history parameters and extrapolating estimates of the extinction probabilities and hazard rates [8, 9, 10, 11]. Moreover, risk assessment of pest outbreaks, as well as colonizing dynamics of invading in quarantine species, can be more precisely predicted in the context of demographic analyses [11, 12, 13].

In addition, because most insect species and related arthropods are considerably shorter-lived than rats and humans and can be easily produced in laboratory conditions, they have often been used for the study of aging. Moreover, such insect species are used to draw universal conclusions of biodemography and aging [8, 14]. Yet, in contrast to mammals, the aging structures in poikilotherms are intimately associated with environmental conditions [15]. Temperature in particular possesses a potent influence on insect population dynamics to such a level that the demographic structure of a particular species may display totally different aging trajectories under different conditions [16, 17].

In survival analysis of human data, when the mortality reaches a peak, past some finite period and then slowly declines, it is appropriate to use a model that has a nonmonotonic failure rate. However, an insect population’s failure rate may exhibit diverse behavior in which the mean residual life displays a reverse fashion with regard to temperature. Actually, there have been very few attempts to probabilistically relate insect aging with respect to temperatures and describe how parameter values of a related aging model may deviate under different temperature regimes.

In this survey, we model the death risk of an insect population system under different conditions using mathematical backbone that is grounded on the general concepts of reliability theory [7, 18, 19, 20, 21, 22]. In reliability theory, the systems were either machine products that were composed of large number of connected components that folded apart after particular time, or, consist of time event sequence data in which the objects (i.e. individuals) have certain probability to fail (death). The failure or death is called an *event*, and the goal is to project or forecast the rate of events for a given population, or, the probability of an event for an individual. In a relative context, we have examined the insect population as a life-system which has as component insect individuals that have a certain likelihood of passing away. However, in this study we are not concerned on how the reliability of the biological system is affected by its components arrangement (e.g. reliability structure at cellular, or, organism level), but emphasize on the population level in which reliability of the system reflects the survival times of the individuals under certain conditions.

Particularly, based on reliability and failure time analysis, the present report is meant to distinguish the major features of survivorship curves and risk rates in moth populations using aging related models and to provide ways in which temperature may enter into consideration in aging models. The scope was to examine whether an exponential-type law of mortality, which is considered as the general rule by many researchers, captures well the insect mortality kinetics under different temperature regimes. This is critical for understanding the mechanisms of insect aging under extreme and favorable conditions because age in poikilotherms is associated with temperature-related increased risk of failure.

Ultimately, the objective is not exclusively to analyze biological data, which provide insights to temperature dependent insect mortality, but likewise, using straightforward modeling techniques, to provide a mathematical background which may conduce to the analysis and understand about aging patterns.

Hence, it is not thought to build models to generate new predictions *per se*, but instead to delineate how the curve parameters of an aging model are altered by ambient temperature. In this setting, the maximum likelihood estimation of the parameters is also examined and it is proved analytically that unique maximum likelihood estimates exist for the parameters. Finally, bivariate interpolations between parameter estimates and temperatures are produced and the probable errors in those analyses are briefly discussed.

## Materials and Methods

### 2.1 Source Material, Rearing Conditions and Collection of Survival Information

A colony of *Anarsia lineatella* Zeller (Lepidoptera: Gelechidae) was established in the Laboratory of Applied Zoology and Parasitology at the Aristotle University of Thessaloniki from field-collected larvae present on infested twigs and peaches in northern Greece. Samples were collected from private peach orchards, and landowners gave permission to collect individuals at these sites. In summary, all field sampling procedures did not involve endangered or protected species and no specific permissions were needed for these actions. The settlement was kept in constant laboratory conditions at 25°C, 65±5% RH and 16:8 h L: D as described in previous works [17, 23]. Five age-synchronized cohorts consisting of 30–50 experimental individuals for each status were established with newly emerged adults and were watched over time. Single-sex populations used for survival studies consisted of males and females in approximately equal ratios. Experimental populations were maintained under controlled conditions at five constant temperature regimes, 15, 20, 25, 30 and 35° C, with constant light cycles (16:8 h L:D) and a relative humidity of 65% [17, 23]. To avoid any irreducible variation in life spans that was not associated with the temperature regimes (i.e., quantitative life trait patterns, confounding uncontrolled environmental variations with treatment of genotype effects) the temperature-related life span treatments were performed simultaneously [24]. As the chords aged, the dead individuals were removed, counted and recorded daily.

### 2.2 Basic Quantities and Functions of Survival Time

The distribution of survival times under different temperature regimes was described by applying the survivorship function [*S(t)*], the respective probability density function [*f(t)*] and the hazard function [*h(t)*] [21].

The survivorship function (or survival function) is defined as the probability that an individual has a survival time *T* longer than *t* [7, 21, 25, 26]:

S(t)=Pr[T>t], (1)

or

S(t)=1−F(t), (2)

where *F(t)* is the standard cumulative distribution function, in the probability theory, with probability density function *f(t)*:

$$F(t)=\mathrm{Pr}[X\leq t]\equiv \int_{0}^{+\infty }f(t)dt\;\forall t\in (0,+\infty ).$$ (3)

Since *F(x)* is continuous:

$$f(t)=\frac{dF(t)}{dt}$$ (4)

and

$$f(t)\geq 0,\forall t\in {ℜ}^{+}.$$

Additionally, the probability of surviving at least time zero is 1 and that of surviving an infinite time is zero, or:

$$\int_{0}^{+\infty }f(t)dt=1.$$ (5)

The cumulative hazard function is defined as:

$$H(t)=\int_{0}^{t}h(x)dx,$$ (6)

having values between zero and infinity and was estimated for the respective survival function as follows [21]:

$$H(t)=-\log_{e}S(t).$$ (7)

Ultimately, the instantaneous (conditional) hazard rate (failure rate or instantaneous risk of failure of reliability theory), is presented as:

$$h(t)=\frac{f(t)}{1-F(t)},$$ (8)

$$h(x)=-\frac{dS_{x}}{S_{x}dx}=-\frac{d[\log_{e}S(x)]}{dx}.$$ (9)

Note that the hazard is not a probability since it counts per time interval *dx* and consist of a rate. Therefore, the hazard function, or force of mortality, gives the risk of dying per unit time during the aging procedure and is limited in terms of the cumulative distribution function and the probability density function [21].

### 2. 3 The Bivariate Weibull Probability Distribution Model

The probability distribution function (*pdf*) of the Weibull model is given as follows [27]:

$$f(x_{i};\alpha ,\beta )=\frac{\beta }{\alpha }{\left(\frac{x_{i}}{\alpha }\right)}^{\left(\beta -1\right)}\exp\{-{\left(\frac{x_{i}}{\alpha }\right)}^{\beta }\},\;x_{i}\geq 0,$$ (10)

where *α>0* is the scale parameter and *β>0* the shape parameter and when *β = 1*, it reduces to the exponential model. Thus, if the quantity *x* is the hazard (e.g., "time-to-failure" according to the reliability theory), the Weibull distribution gives a distribution for which the hazard rate is proportional to a power of time [28].

The mean and variance of the Weibull distribution, having parameters *α* and *β*, are, respectively:

$$\mu =\alpha \{G(1+\frac{1}{\beta })\}$$ (11a)

And

$$\mathrm{var}=\alpha^{2}\{G(1+\frac{1}{\beta })-G{\left(1+\frac{1}{\beta }\right)}^{2}\},$$ (11b)

where G states for the gamma function:

$$G(x)=\int_{0}^{\infty }t^{x-1}e^{-t}dt,\;x\in (0,\infty )$$ (12)

The mean of and variance of the Weibull distribution, having parameters specified by *α* and *β*, was generated as an expansion to a constant matrix with the same dimensions for both parameters [29, 30].

The survival function is given as:

$$S(x_{i};\alpha ,\beta )=1-\{1-\exp{\left[-(\frac{x_{i}}{\alpha })\right]}^{\beta }\}$$ (13)

The cumulative survivorship distribution function (*cdf*) of the Weibull model is:

$$F(x_{i};\alpha ,\beta )=1-\exp{\left[-\frac{x_{i}}{\alpha }\right]}^{\beta }$$ (14)

and the hazard is:

$$h(x_{i};\alpha ,\beta )=(\frac{\beta }{\alpha }){\left(\frac{x_{i}}{a}\right)}^{\left(\beta -1\right)},$$ (15)

where *β* states for the shape parameter.

Moreover, to provide a means of inference we generated the *log* of the Weibull hazard, which is a linear function of *log* time with constant $\log(\frac{\beta }{\alpha })$ and slope: *β*−1. Thus, the hazard was generated in respect to each of the different temperature regimes and is rising if *β*>1, is constant if *β* = 1, and is declining if *β*<1.

### 2.5 Maximum Likelihood Parameter Estimation

If we denote with *x*_1_,*x*_2_,…,*x*_*n*_ the random sample size *n*, then the probability of obtaining the particular sample is as follows:

$$P(X_{1}=x_{1},X_{2}=x_{2},\ldots ,X_{n}=x_{n})=P(X_{1}=x_{1})P(X_{2}=x_{2})\ldots P(X_{n}=x_{n})=\prod_{i=1}^{n}P(X_{i}=x_{i})$$ (16)

The two-parameter probability model is denoted as *f*(*x*;*α*,*β*) and we replaced the generic probability terms with the proposed mode so that [31]:

$$\prod_{i=1}^{n}f(x_{i};\alpha ,\beta )=\prod_{i=1}^{n}P_{\alpha ,\beta }(X_{i}=x_{i})=L(\alpha ,\beta ;x_{1},x_{2},\ldots ,x_{n}).$$ (17)

To date, the expression on the left side is referred to as the likelihood, *L*, of the survivorship data under the proposed model (Weibull 1951). Since the logarithm is a monotone increasing function, it is convenient to work with the log-likelihoods and negative log-likelihoods, which are as follows:

$$\log L(\alpha ,\beta ;x_{1},x_{2},\ldots ,x_{n})=\sum_{i=1}^{n}\log[f(x_{i};\alpha ,\beta )]$$ (18)

We can now proceed to the estimation of the maximum likelihood estimates (MLEs) of the parameters *α* and *β*, which make the likelihood as large as possible; i.e., maximize the probability of the observed data under the resulting model we applied, local maxima occur at points where the first derivative is equal to zero [32]. In the bivariate Weibull model, this involves the estimation of the derivative of the log-likelihood with respect to each of the two parameters. To do this, we put each of the derivatives equal to zero and solve the following scheme (e.g., Score gradient vector):

$$h(a,\beta )=\left[\begin{array}{l} \frac{\partial }{\partial \alpha }\log L(\alpha ,\beta ;x_{1},x_{2},\ldots ,x_{n})\\ \frac{\partial }{\partial \beta }\log L(\alpha ,\beta ;x_{1},x_{2},\ldots ,x_{n}) \end{array}\right].$$ (19)

The matrix of second partial derivatives of the log-likelihood is called the Hessian matrix:

$$H(a,\beta )=\left[\begin{array}{l} \frac{\partial^{2}}{\partial \alpha^{2}}\log L(\alpha ,\beta )\;\frac{\partial^{2}}{\partial \alpha \partial \beta^{}}\log L(\alpha ,\beta )\\ \frac{\partial }{\partial \alpha \partial \beta }\log L(\alpha ,\beta )\;\frac{\partial^{2}}{\partial \beta^{2}}\log L(\alpha ,\beta ) \end{array}\right]$$ (20)

and was estimated to obtain MLE according to the *Newton-Raphson* optimization method, which uses both the h vector and the H matrix as follows:

$$\theta =\left[\begin{array}{l} \alpha \\ \beta \end{array}\right]$$ (21)

and

$$\theta_{\kappa +1}=\theta_{k}-H^{-1}(\theta_{k})g(\theta_{k}).$$ (22)

In the above recurrence relation, ***H*** ^*-1*^ denotes the inverse of the Hessian matrix and allows using new entry values for the two distribution parameters to obtain updated values for each step ahead, which better represents the true roots of derivatives [33, 34, 35].

Moreover, the logarithm transforms the product of potentially small likelihoods into a sum of logs, which is easier to distinguish from *0* in the computation. Therefore, for convenience and to project the negative log likelihood 3D surface, the following negative log-likelihood function was estimated as [36]:

$$(-\log L)=-\log\prod_{i=1}^{n}f(\alpha ,\beta ;x_{i})=-\sum_{i=1}^{n}\log f(\alpha ,\beta ;x_{i}).$$ (23)

The parametric family of distributions was specified first by using a fixed scale parameter, *α*, over different values of the shape parameter, *β* and to compute the likelihoods for the reference temperature (i.e., optimum temperature of insect development at 25°C) using R [37].

The MatLab [38] Newton-Raphson optimization algorithm was used to generate a parametric family of distributions and to estimate the negative log likelihoods using gradient vectors and Hessian matrix.

## Results and Discussion

**Fig 1:**
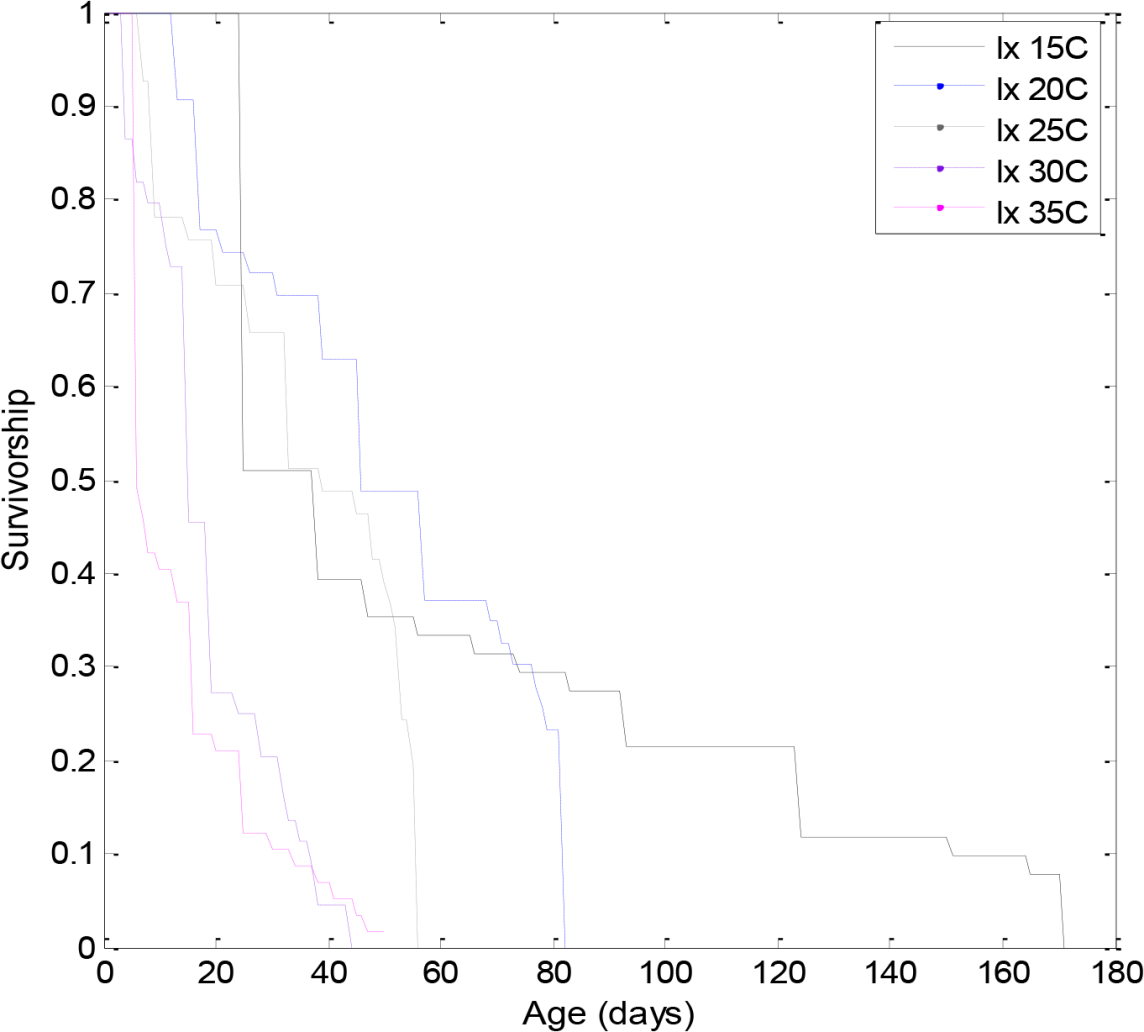
Graphical assessment of actuarial survivorship estimators of the proportional hazard assumptions (age related survivorship) separately for each temperature (pooled data: 15, 20, 25, 30 and 35°C).

**Fig 2:**
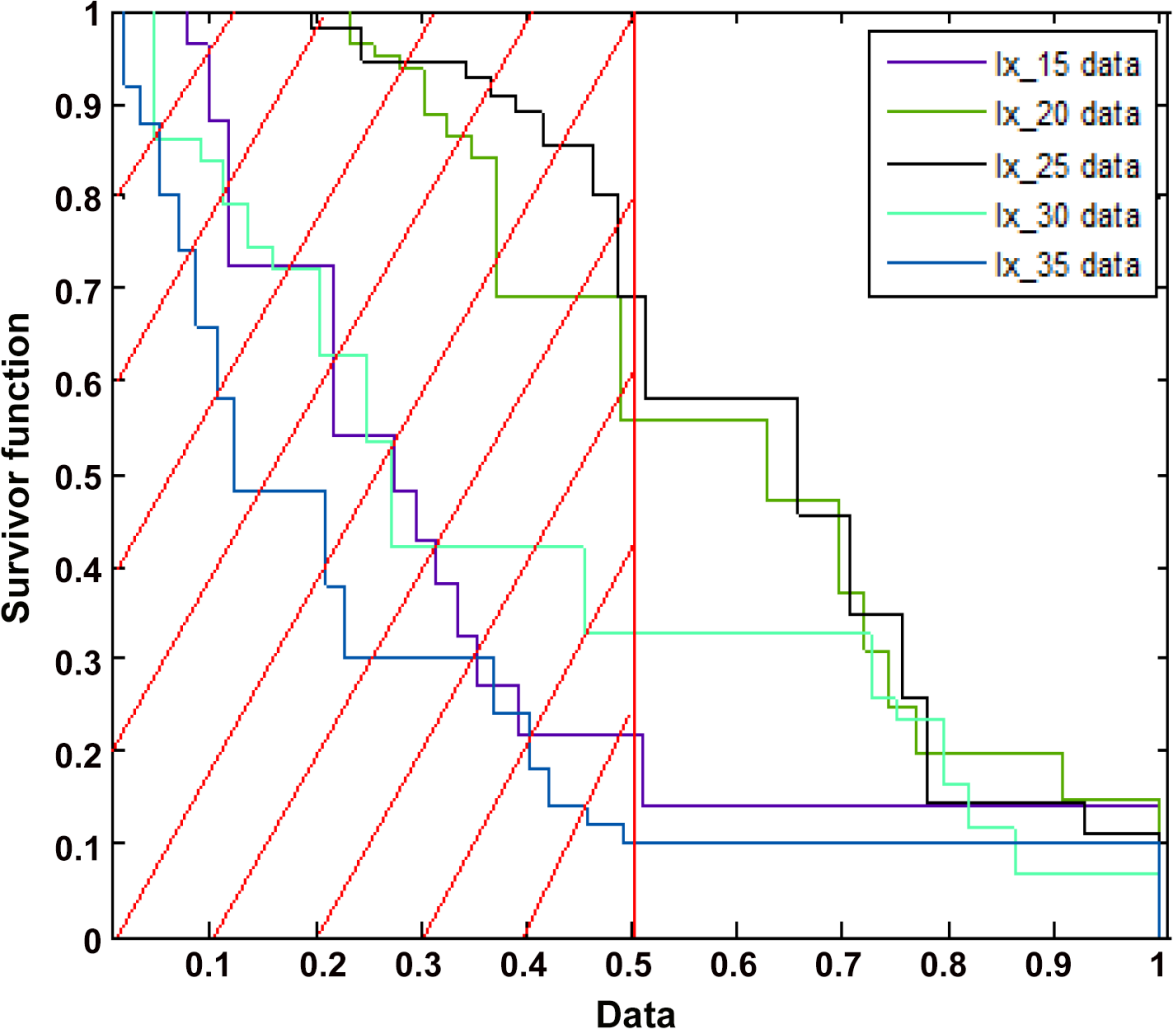
Graphical assessment of actuarial survivorship estimators of the proportional hazard assumptions separately for each temperature (data: 15, 20, 25, 30 and 35°C). Data are sliced into regions in order to emphasize the effect of temperature on failure times as observed in 50% of the female individuals).

Fig 1 depicts the daily survivorship throughout the life of the adult moths in respect to each of the five temperature regimes tested (S1 File), while Fig 2 depicts the probability-related empirical survivorship function (S2 File). Although insect longevity was significantly affected (i.e., lower temperatures were associated to higher life spans and *vice versa*), the form of the survivorship functions appears quite similar for populations that were maintained throughout their lives at the extreme temperatures (i.e., 15 and 35°C). In other words, when longevity was regarded as a failure process it was affected in the same manner under harsh conditions (Fig 2). In particular, in the extremes temperatures we have observed the same effects in the aging process of the insect population, although individuals were clearly affected in a different manner under 15 and 35°C (e.g. showing longevities five times different between treatments). Thus, extreme climatic events, may cause the same effects in the insect aging process.

Survivorship has a key role in the life table analysis (e.g. [17]). However, age-related survivorship does not offer an illustrative evident to interpret probabilistic insect aging rate under certain weather conditions. Universally, the survivorship patterns may constantly distribute over a mean for warm blood species and not simply for insects and related arthropods. Because poikilothermic development may be considered as a macroscopic revelation of enzyme reactions [15] in which temperature exerts a catalytic effect at a molecular level, the temperature-related aging responses that were observed could be explained to some extent by the detrimental effect of temperature on enzyme structure and by its catalytic effects on physiological functions. However, other factors such as temperature-related gene expression should not be excluded, considering that genotype-environment interaction for quantitative trait loci have been reported to affect longevity in *Drosophila melanogaster* [39, 40].

**Fig 3:**
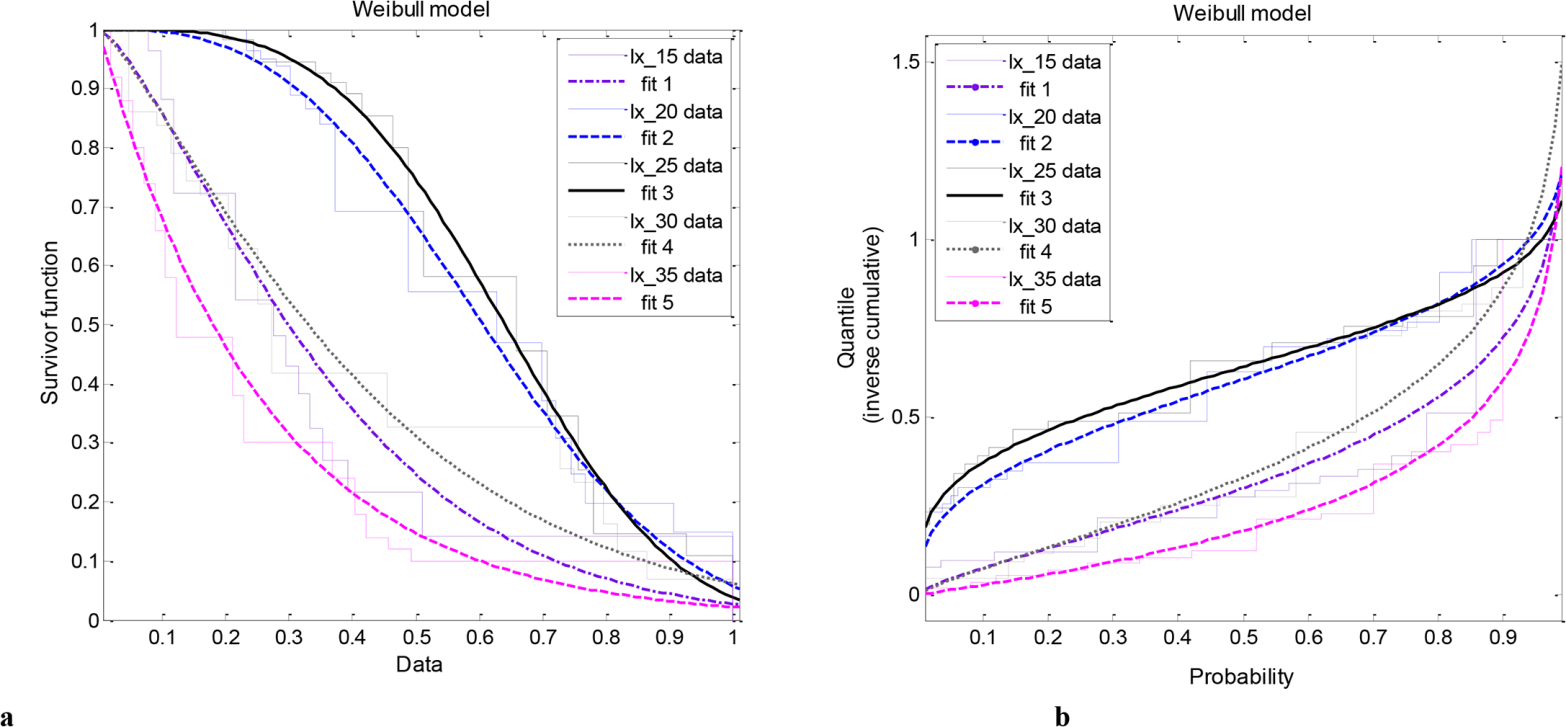
Temperature related model performance of the bivariate Weibull survivorship model (a) and the respective quintile probability plot (b).

The aging process and forecasted decline of populations according to the Weibull model is illustrated in Fig 3. The properties of the survivorship functions, for each temperature regime were well-captured according to the bivariate Weibull distribution model that was applied to the data (Fig 3A). It is apparent that the shape of the curve (e.g., parameters) was dominated by the effect of temperature and the curves can be clustered in two families: one family of curves that describe the insect failure rate under extreme conditions and are more or less similar to the exponential decay model and a second, that describes insect aging—death failure for optimal temperature conditions. These properties may also be summarized by the cumulative quintile-probability plots (Fig 3B), and different shapes for each temperature case may be explained due to the fact the extreme temperatures may be considered as the most probable abiotic mortality factor for insects and related arthropods. Therefore, in contrast to warm blood species, in which death rate is characterized by a particular-stable function (often Gompertz) [18, 41], the probability of dying in poikilotherms may deviate through development in field conditions. For instance, for temperate climates the income of low temperatures during the end of seasons may considerably affect insect aging.

The parameters of the Weibull survivorship model are given in Table 1. The temperature-related model contains all necessary information with a minimum number of parameters, while conflicts and similarities in parameter values summarize in brief the analogies of the generated bivariate Weibull model for each event.

**Table 1:** Mean, variance, log likelihoods and parameters of the Weibull survivorship model in respect to five temperature regimes.

Temperature	15°C	20°C	25°C	30°C	35°C
Mean	0.3578	0.6149	0.6401	0.4117	0.26
Variance	0.0703	0.0558	0.0417	0.1097	0.068
Log Likelihoods	19.0461	2.789	9.3491	-3.3436	17.288
*α(Std*. *Err*.*)*	0.391 (0.0233)	0.6904 (0.0287)	0.7118 (0.0291)	0.442 (0.0568)	0.2594 (0.0391)
*β(Std*. *Err*.*)*	1.3645 (0.0772)	2.82 (0.25)	3.4663 (0.3686)	0.0027 (0.0237)	0.9926 (0.1058)

**Fig 4:**
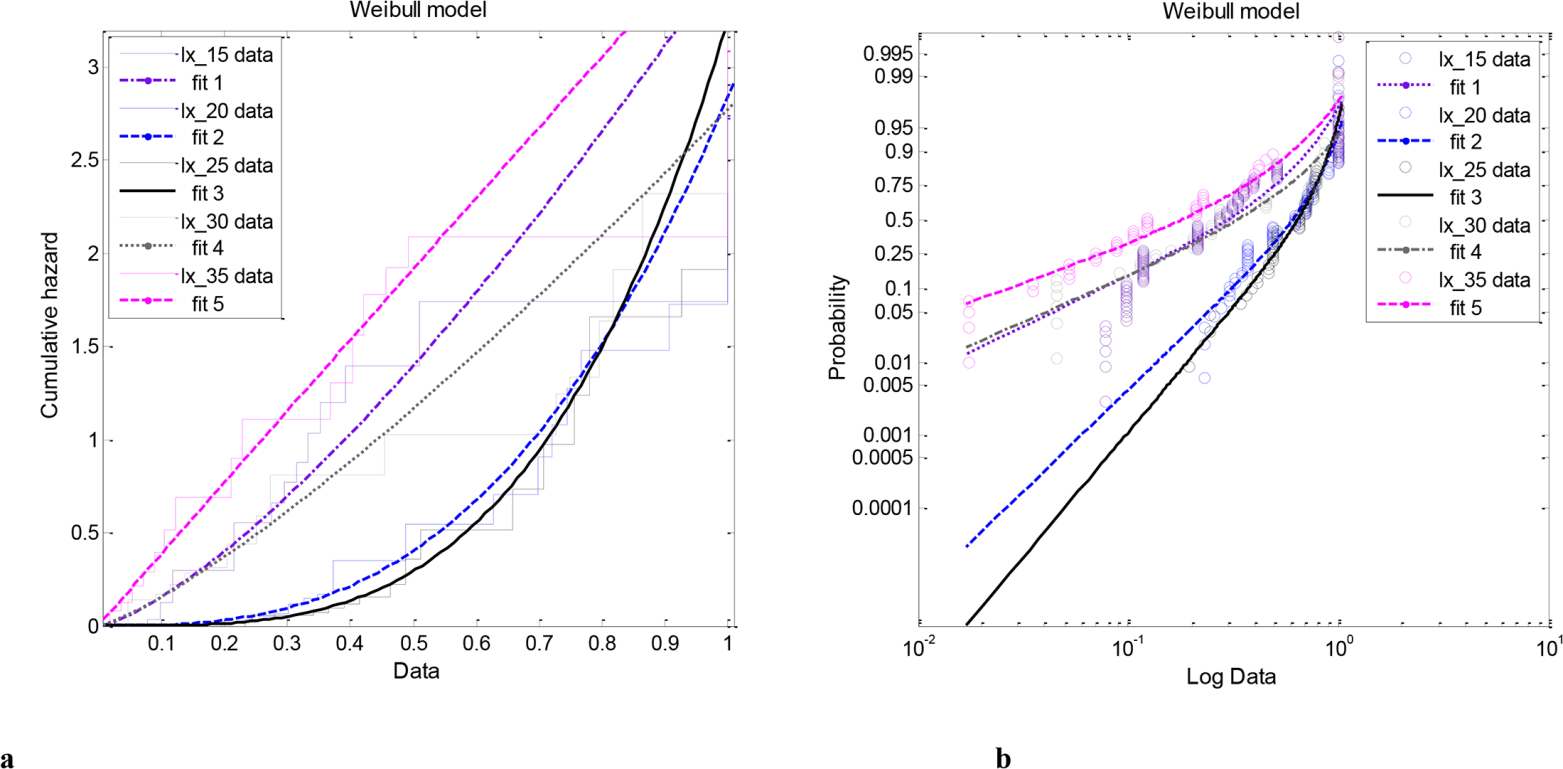
Temperature related model performance of the bivariate Weibull cumulative hazard model (a) and the respective log transformed probability plot (b).

The hazard was calculated for each cohort using the respective Weibull model and the resolutions are summarized in Fig 4A. It is obvious that dramatic change in mortality had occurred when populations were maintained at extreme temperatures. Particularly, the failure rate (i.e., hazard rate or force of mortality) was accelerated in populations that were kept under the extreme temperatures by the same, linear, manner, in contrast with the intermediate temperature regimes in which the hazard rate increased exponentially at older ages.

In reliability theory, the increasing failure rate is an intuitive concept caused by components wearing out and describes a phenomenon where the probability of life event at the fixed time intervals in the future increases over time [31]. However, in the case where populations were kept at optimum temperatures earlier failures were fewer and failure rates increased exponentially with age, in contrast with failures in populations that were kept at the extremes in which rates increased linearly with age.

Fig 4B depicts the convergence rates of mortality at each temperature according to the log-hazard probability functions. The failure rates are plotted along a log scale as a function of age. Of note, the age-specific probability of death grew monotonically with age up to an advanced age and then slightly leveled off. Nevertheless, it is quite interesting, again, that the failure kinetics of the populations maintained at extreme temperature demonstrated similar non—exponential aging patterns, while individuals did not died more frequently as they grow older in contrast to those that were preserved at the favorable for growth and development temperature regimes.

Although mortality patterns of the population are the result of individual deaths, if we regard the laboratory populations under a holistic perspective and as life system, we can deduce some interesting remarks. From a reliability theory standpoint for instance, constant failure rates at the extremes indicate that the system (population) is capable to eliminate earlier failure and/or to keep later failure rates constant. Because biological aging in poikilotherms is related to intrinsic causes (i.e. physiological and enzymatic reaction; [15] and references), which are adjusted in response to the extrinsic temperature mortality factors, we may assume that the extreme temperatures can effect physiological functions, resulting to constant increases in failure rates.

Therefore, in contrast to the survivorship models, both the hazard rate and the related semi log- probability plots may useful in defining periods of mortality that are leveling off.

In other insect species, including fruit flies, *Drosophila melanogaster*, nematodes, mosquitoes, human lice, flour beetles and mice, the increment in age-specific mortality is reported to follow the exponential law (i.e., Gompertzian), while technical devices usually fail according to the *Weibull* (*power*) law [41, 42]. Nevertheless, there are also some exclusions to the Gompertz law of mortality, where the organisms die according to the Weibull (power) law and thus display more or less a constant chance of dying at all ages [43,44]. Even so, the response to the question of why species display differently aging patterns under certain conditions remains a heavy challenge for many hypotheses of aging and longevity [45, 46]. Since the failure rate had different shapes in respect to temperatures, the current results provide some clues to the striking patterns of the population failure rate in poikilotherms.

**Fig 5:**
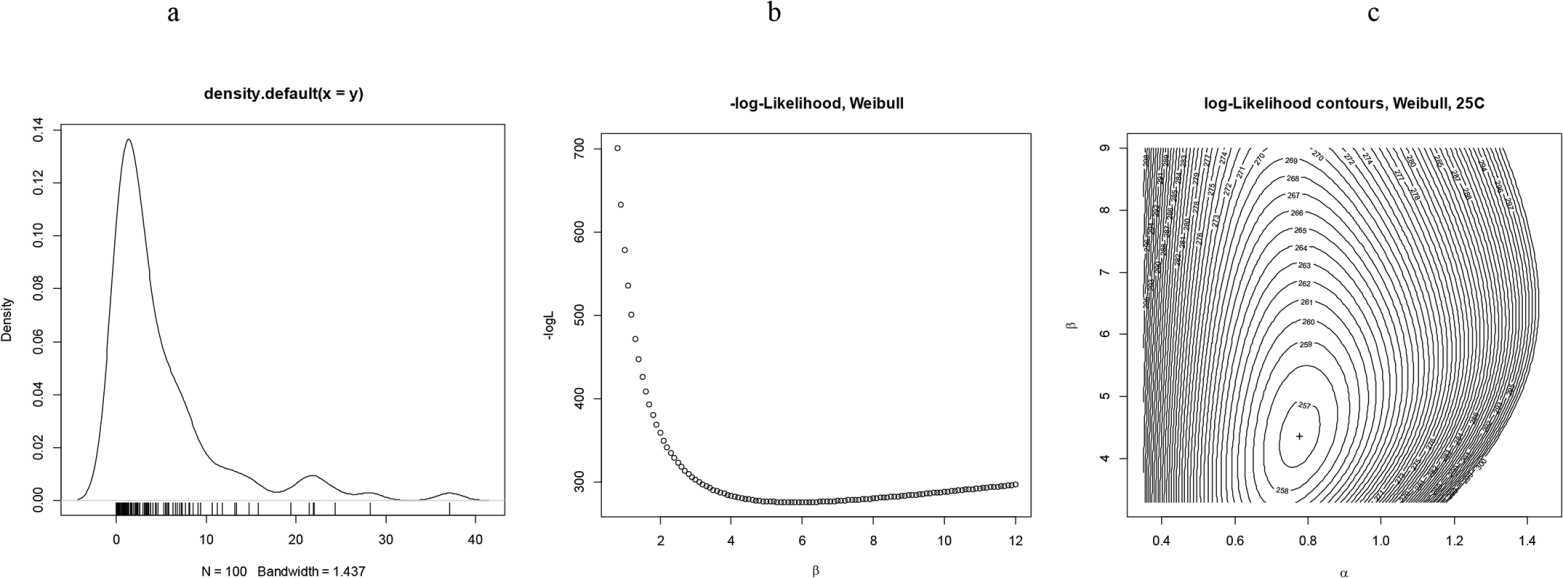
Temperature related trends towards increased mortality with increasing age and respective Cumulative hazard (a) and the probability of dying scaled log transformed the size of the population per unit of time (b). The application is done by examining the standard hazard processes used to describe the age-related risk of death using the Weibull function. Notice that populations that were maintained at the extreme temperatures (15, 30 and 35°C) have a constant—linear increase in failure rate (risk of death), while populations that were maintained at optimum conditions (20 and 25°C) have accelerated with age, exponential increase, in failure rate.

Fig 5A illustrates the random simulations of the mortality patterns according to the Weibull probability density functions using the parameter values that were calculated for the populations that were maintained at 25°C, which is the optimum temperature of species evolution. Fig 5B are the results of the negative log-likelihoods of the shape parameter *β* in relation to a fixed value of the scale parameter *α*. Fig 5C is a log-likelihood contour which depicts the bivariate minimum values of the likelihood with regard to different parameter combinations.

**Fig 6:**
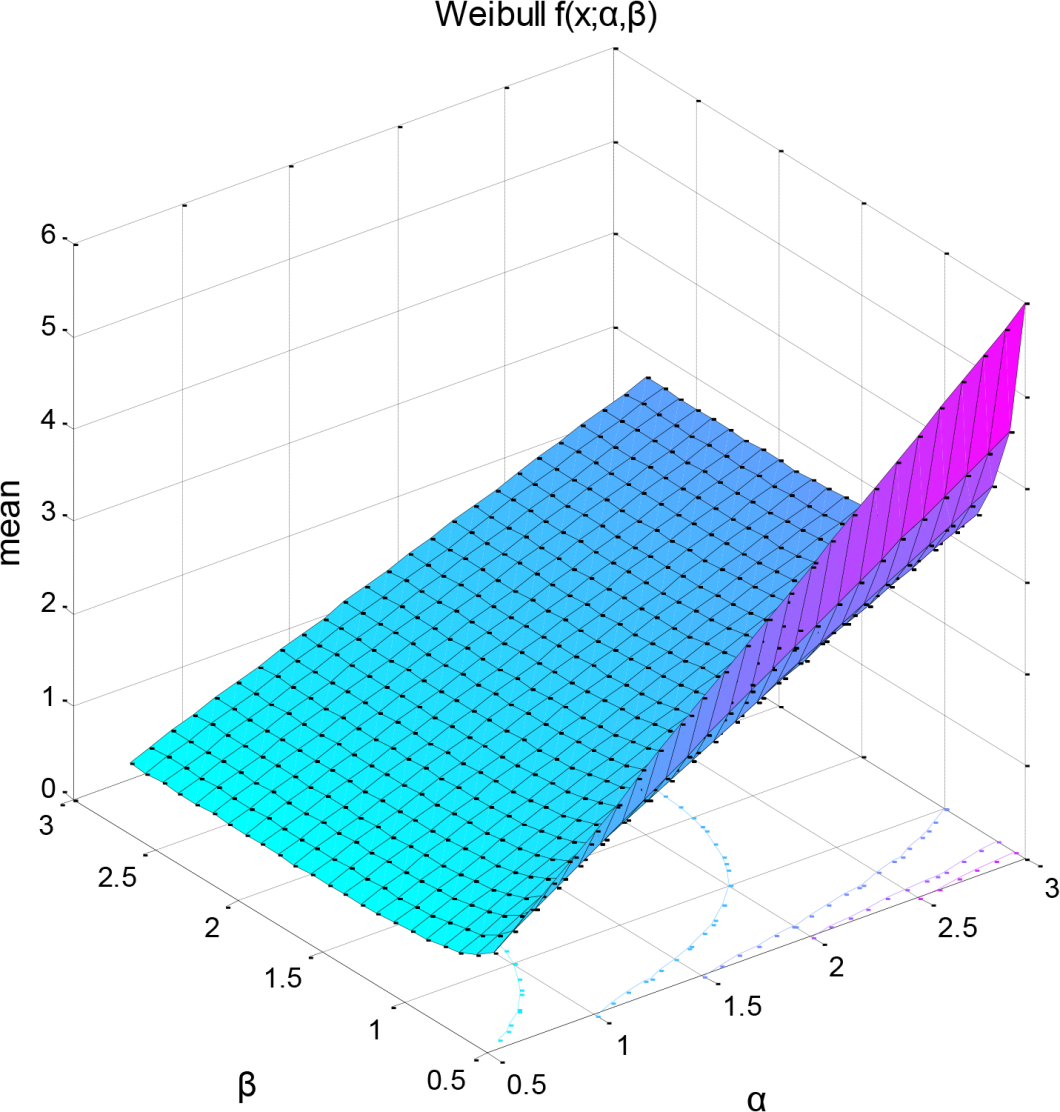
(a): Representative simulated *pdf* by the Weibull model using the standard shape and scale parameters of populations maintained at 25°C (optimum temperature of development). b) Monovariate examination of the profile likelihood of the shape parameter (*0<β<12*) for fixed scale parameter (*α* = *0.7*) (the MLEs maximize *logL* (*f, x_i_, a*) over all possible *β* over the independent aging variable *x_i_*) (c) Bivariate examination of the profile likelihood for both shape and scale parameters and parametric family of distributions specified by its pdf *f* (*x, α, |b*), the simulation results of the Newton-Raphson algorithm are: Local minimum: 60.92, score gradient vector: $h(a,\beta )=\left[\begin{array}{l} 2065.761\;-42.1044\\ -42.104\;9.6543 \end{array}\right]$ and Hessian matrix: $\text{H}(a,\beta )=\left[\begin{array}{l} 0.00053131\;0.0023172\\ 0.00231717\;0.1136870 \end{array}\right]$.

**Fig 7:**
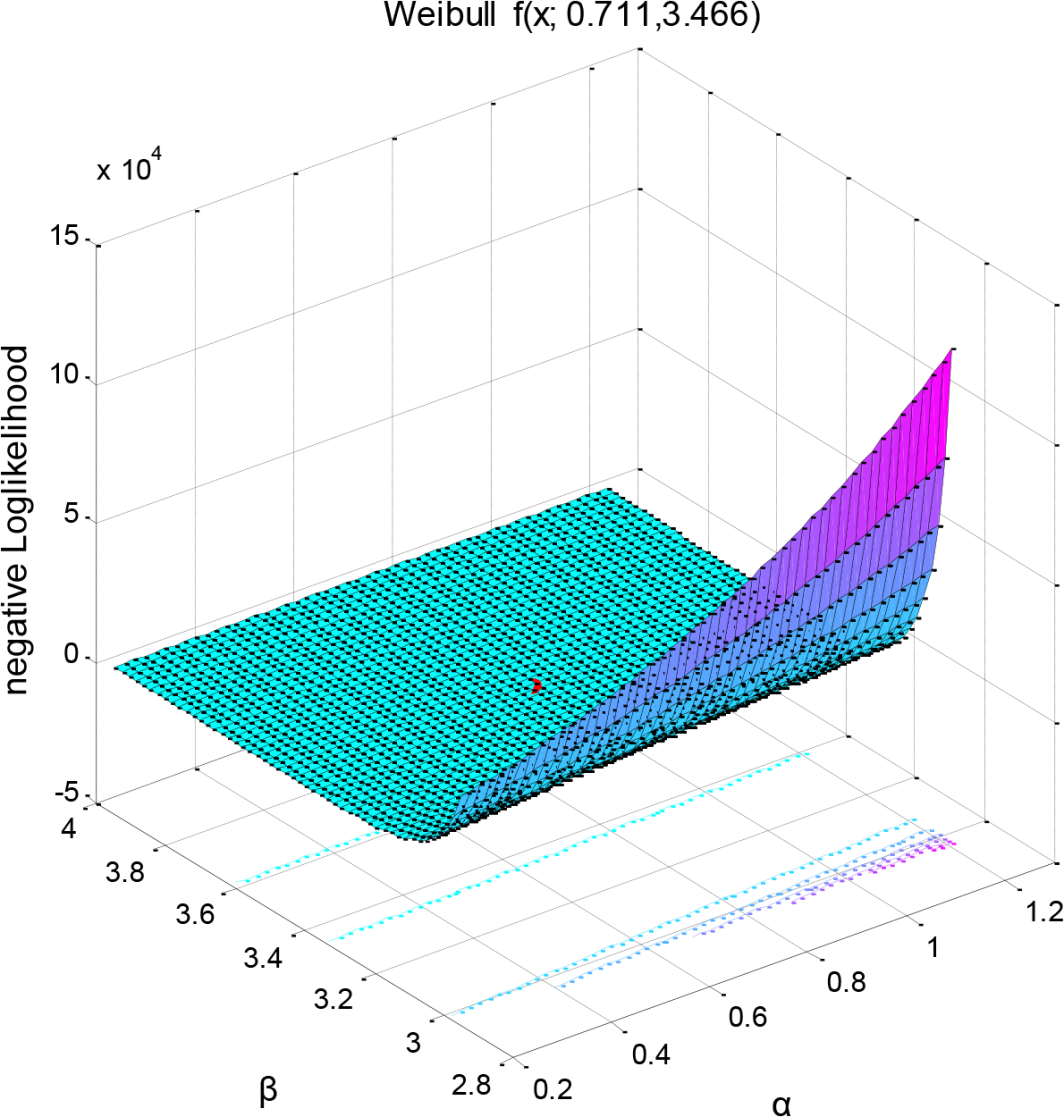
Bivariate 3D interpolation of the mean of the Weibull distribution as a function of its two distribution parameters (mean = 0. 6394, var = 0.0417) (left) and negative log likelihood surface of a parametric family of distributions (right). The negative log-likelihood function has as input arguments the combination of the two parameter values and was used to return the return the negative of this sum. Here the optimization algorithm to which the values are passed searched for minima rather than maxima. Local minimum is printed in red and was estimated using: $\left(-\log L)=-\log\prod_{i=1}^{n}f(x_{i};a,\beta )=-\sum_{\iota =1}^{n}\log f(x_{i};a,\beta \right)$.

Fig 6 depicts the mean of the Weibull distribution as a subroutine of its two distribution parameters (mean for *μ = 0*.*6394*, *var = 0*.*0417*) and Fig 7 shows the related negative log-likelihood surface of a parametric family of distributions having a combination of the two parameter values as the input arguments. The MLE optimization process produced an approximate assessment of the parameter values, which may provide explanation of the way in which species life span and age-related probability of dying may be affected by environmental conditions. The negative MLE surface projections are found by solving the system of the two non-linear equations according to the Newton-Raphson optimization algorithm and return the minima rather than maxima as for positive MLE.

Noteworthy to state is that the MLE optimization process that was applied produced not only an approximate assessment of the Weibull model parameter values, but also the ‘best’ solutions in respect to the different parameter combinations. To date, one important challenge in MLE, is the specification of likelihood functions and the selection of a set of values (point estimates) which fit to data and eventually, using suitable algorithms to avoid instabilities of the estimated coefficients. In addition, the number of objective function evaluations (approximations), which have been illustrated, captures most of the computational cost of the method we have applied and an added value of these projections is that they can be applied to compare parameter performance with other MLE algorithms [47, 48].

**Fig 8:**
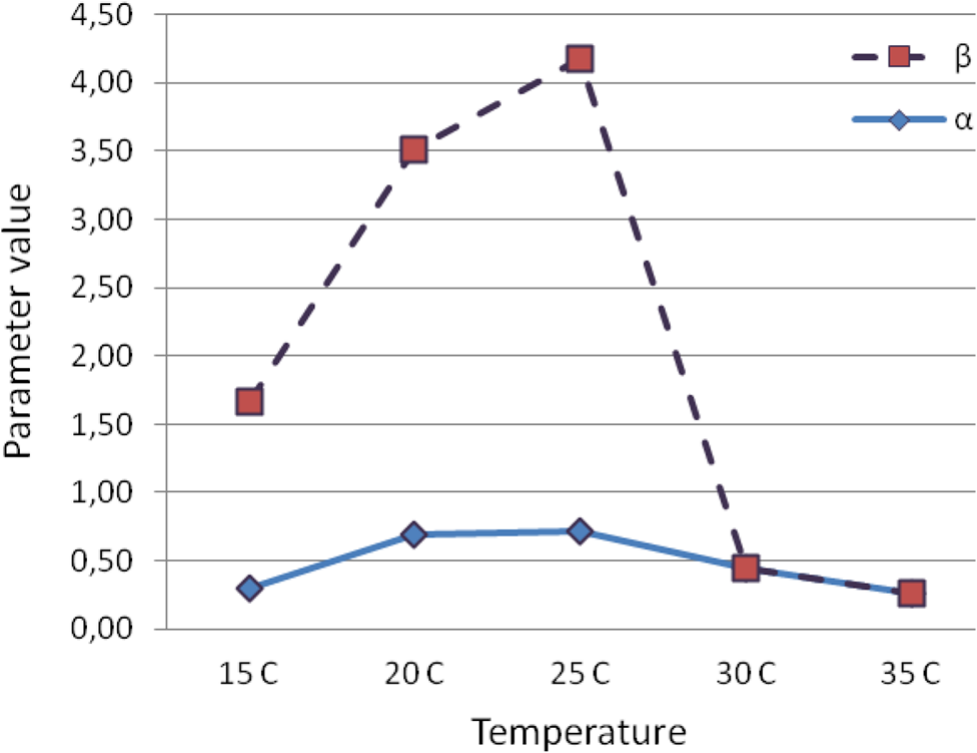
General overview of the temperature effects on the bivariate parameters of the Weibull pdf model *f(x;α*,*β)* (α: scale parameter and β: shape parameter).

Nevertheless, the critical issue raised by this analysis is whether the parameters of the Weibull model would demonstrate the same features with regard to temperature as shown in Fig 8. If so, it may then be possible to use surface roughness measurements straightforwardly to determine the point of deviation from a standard (optimum) condition. For example, the MLE estimates of the scale parameter vary considerably compared with the shape parameter (Fig 8) and the comparative magnitudes of the *β* values do exhibit high differences. However, it should also be outlined that parameter differences should not be viewed strictly as quantitative differences, but as qualitative, because a slight variety in a parameter value may significantly affect the build of the model.

Many efforts have been formed to find mathematical patterns that will summarize the direction in which the probability of dying depends on age, and such formulas have been used as potential applications [44, 49, 50, 51]. In this setting, the current generated models may be helpful in the projection of population numbers, in the aid of the insect population actuarial work, and, particularly, in the detection of how lifespan is affected by abrupt environmental conditions. In particular, the current actuarial analysis provides a detailed description of the nature of the linkage between the dynamics of population’s mortality and temperature. It is rather amazing that based only on knowledge of survival functions and hazard rate models, the current analysis allows a sensible prediction of the increase in subsequent death rates at the population level tier with regard to temperature.

## Concluding Remarks

In this work we have established a parallelism between insect population and a reliability system, by considering the insect cohorts as a life system having as items individuals that pass away. Particularly, although the mortality patterns of the population are the result of individual deaths, if we consider the laboratory cohorts as an isolated life system (i.e. a population in a constant environment), we may further proceed to its reliability analysis. The approach applies statistical engineering methods, in which time represent survival time of individuals to predict population failure and thus draws inferences about population aging under certain conditions. Using reliability analysis the insect population extrinsic mortality in nature is simulated in the laboratory by controlled causes of mortality to manifest how temperature regulates the rate of aging.

The study reveals for the first time that the Weibull function describes contrasting biological causes of demographic aging for populations maintained at different temperature regimens. The terms describing increasing mortality with age may be interpreted as multiplicative at optimum temperatures and additive at the extremes [52]. From a reliability standpoint, these trends could result from an increase in the vulnerability of individuals to external related (i.e. abiotic stress) and intrinsic (genetic) related causes, respectively. Comparisons of the rate of aging under different conditions, should allow to distinguish on biological grounds the causes of mortality and has meaning for the way mortality relates the process of normal aging in biological systems [53, 54, 55]. For example, in genetically heterogeneous populations of the nematode *Caenorhabditis elegans*, constant age-specific mortality results from genetic heterogeneity [56]. Nevertheless, due to the absence of similar trials we cannot determine the absolute cause of mortality for each temperature which explains the different patterns of age-dependent increase in mortality (i.e. external vs intrinsic causes).

Overall, the diligence of the Weibull model exhibited good fit to the survivorship data and can improve the predictive capabilities of modeling mortality in poikilotherms under variable environments. A practical advantage of the described method of modeling mortality kinetics is its descriptive power to distinguish diverse aging patterns in regard to different conditions. From an empirical standpoint the function fits age-death equally well and this is a general characteristic of the Weibull model, which is applied on other species as well [57, 58]. Thus, insect population mortality kinetics are accurately described and in a way that the effect of an external factor (temperature), can be constituted by means of changes in parameter estimates (scale), the relative position lines (model projections) and slopes of the probability plots (shape parameter). As, for example, the exemplification of the relation between temperature and parameter estimates to calculate the degree of departure from the optimum conditions.

Founded on the MLE of parameters and the related model extrapolation, life measures can be directly obtained using the appropriate equations for each condition. The latter shows an overview of the effects of temperature on the parameters of the bivariate Weibull model and may use to interpret each scale and parameter combination. The values of the shape parameter, for instance, differentiate dramatically for each case, suggesting that there is no general function which can describe insect aging in all circumstances. Another empirical observation is that in most cases the higher values of the shape parameters are compensated by lower values in the scale parameters. However, when the model was extrapolated by the log–transformed hazard, the particular combination of parameters may be considered as a type of coordinates for mortality convergence since all mortality trajectories finally fall into a single point area.

Biologically, the model states that under extreme temperatures each individual drifts toward death in a linear fashion and has a constant chance of passing away, while on the other hand, under optimum temperature conditions, the likelihood of death may increase as age advances. The finding that the force of mortality levels off beyond a certain ages by different manners, may appear surprising, because, traditionally, it was thought as rule that in an aging population force of mortality increases exponentially until all individuals have died. To boot, the aging patterns we have observed are different compared to well established deviations in hazard rates that have observed constant hazard rates at advanced ages [58, 59, 60]. Especially, at the optimum temperature conditions the hazard rate accelerates at older ages, while at the extremes, it is stabilized increasing linear instead curving and possibly plateauing (i.e. late life mortality deceleration). Nevertheless, although deviations from exponential growth rates have been noted in other insects, studies with mammals (i.e. rodents) have found varying conclusions [61 and references]. Related deviations from the exponential rule of longevity occurs also in failure rates of manufactured products [62].

Even so, the current results indicate that this rule is valid exclusively along certain terms rather than the population is maintained under favorable-optimum temperatures. Furthermore, counting that the existence of alternating temperatures is more probable in reality, we may expect deviations from exponential demographic aging in insect populations under natural conditions. Actually, under field conditions ecological forces, predators, matting success etc. may cause cumulative damage that increases age-related mortality rates [63, 64].

Although it is hard to explain why a population generates different future mortalities, between extreme and favorable conditions, the current work discovers the extent to which temperature could alter population aging and may add to the related aging theories and ecological interpretations (i.e. refer to [41, 65, 66], for a thorough review on theories and related modeling approaches). We may argue, for instance, that the different accelerations in the velocity of age rates may reflect the tensions caused by temperature on the physiological functioning of insects and their related enzyme reactions. Moreover, the fact that the terms increasing mortality with age are additive in the extreme conditions could result from an increase in the vulnerability of individuals due to extreme conditions, while the multiplicative age patterns at the optimum condition, may be linked to physiological damage and which is more possible as age advances. Therefore, changes in population aging may be reflecting the differences in the individual mortality rate of extreme temperatures.

Ultimately, the current study may provide a secure and comprehensive actuarial foundation for temperature-related mortality analysis since it incorporates an interdisciplinary perspective on demographic concepts and actuarial techniques into the entomological and life science literature.

## Supporting Information

S1 File Actuarial survivorship of insect population cohorts (Kx). (TXT)

S2 File Proportional survivorship of insect population cohorts (lx). (TXT)
