# Correction: Do Insect Populations Die at Constant Rates as They Become Older? Contrasting Demographic Failure Kinetics with Respect to Temperature According to the Weibull Model

**DOI:** 10.1371/journal.pone.0139526

**Published:** 2015-09-25

**Authors:** Petros Damos, Polyxeni Soulopoulou

There are errors in the captions for Figs 4–7. Please see the complete, correct captions here.

**Fig 4:**
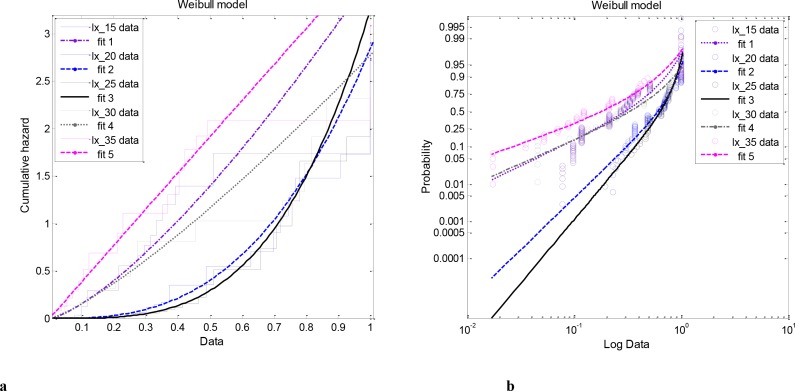
Temperature related trends towards increased mortality with increasing age and respective Cumulative hazard (a) and the probability of dying scaled log transformed the size of the population per unit of time (b). The application is done by examining the standard hazard processes used to describe the age-related risk of death using the Weibull function. Notice that populations that were maintained at the extreme temperatures (15, 30 and 35°C) have a constant—linear increase in failure rate (risk of death), while populations that were maintained at optimum conditions (20 and 25°C) have accelerated with age, exponential increase, in failure rate.

**Fig 5:**
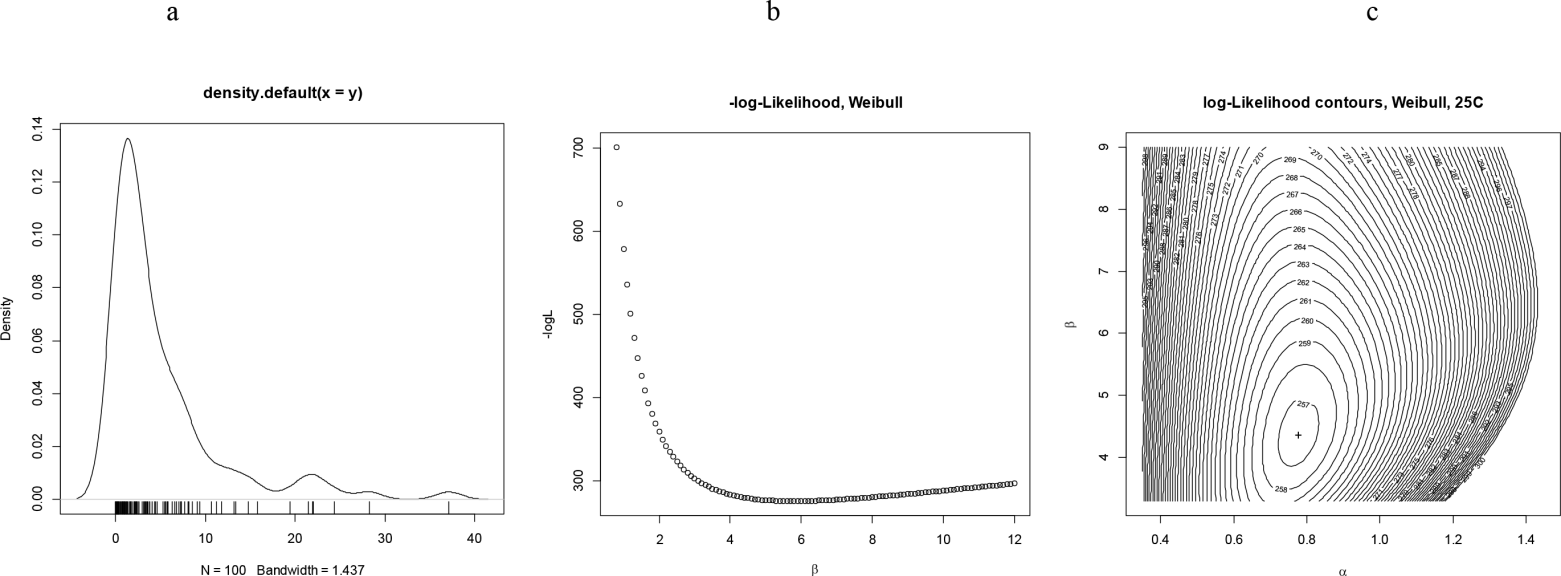
(a): Representative simulated *pdf* by the Weibull model using the standard shape and scale parameters of populations maintained at 25°C (optimum temperature of development). b) Monovariate examination of the profile likelihood of the shape parameter (*0<β<12*) for fixed scale parameter (*α = 0.7*) (the MLEs maximize *logL* (*f, xi, a*) over all possible *β* over the independent aging variable *xi*) (c) Bivariate examination of the profile likelihood for both shape and scale parameters and parametric family of distributions specified by its pdf f (*x, α, |b*), the simulation results of the Newton-Raphson algorithm are: Local minimum: 60.92, score gradient vector: $\text{h}(a,\beta )=\left[\begin{array}{cc} 2065.761 & -42.1044\\ -42.104 & 9.6543 \end{array}\right]$ and Hessian matrix: $\text{H}(a,\beta )=\left[\begin{array}{cc} 0.00053131 & 0.0023172\\ 0.00231717 & 0.1136870 \end{array}\right]$.

**Fig 6:**
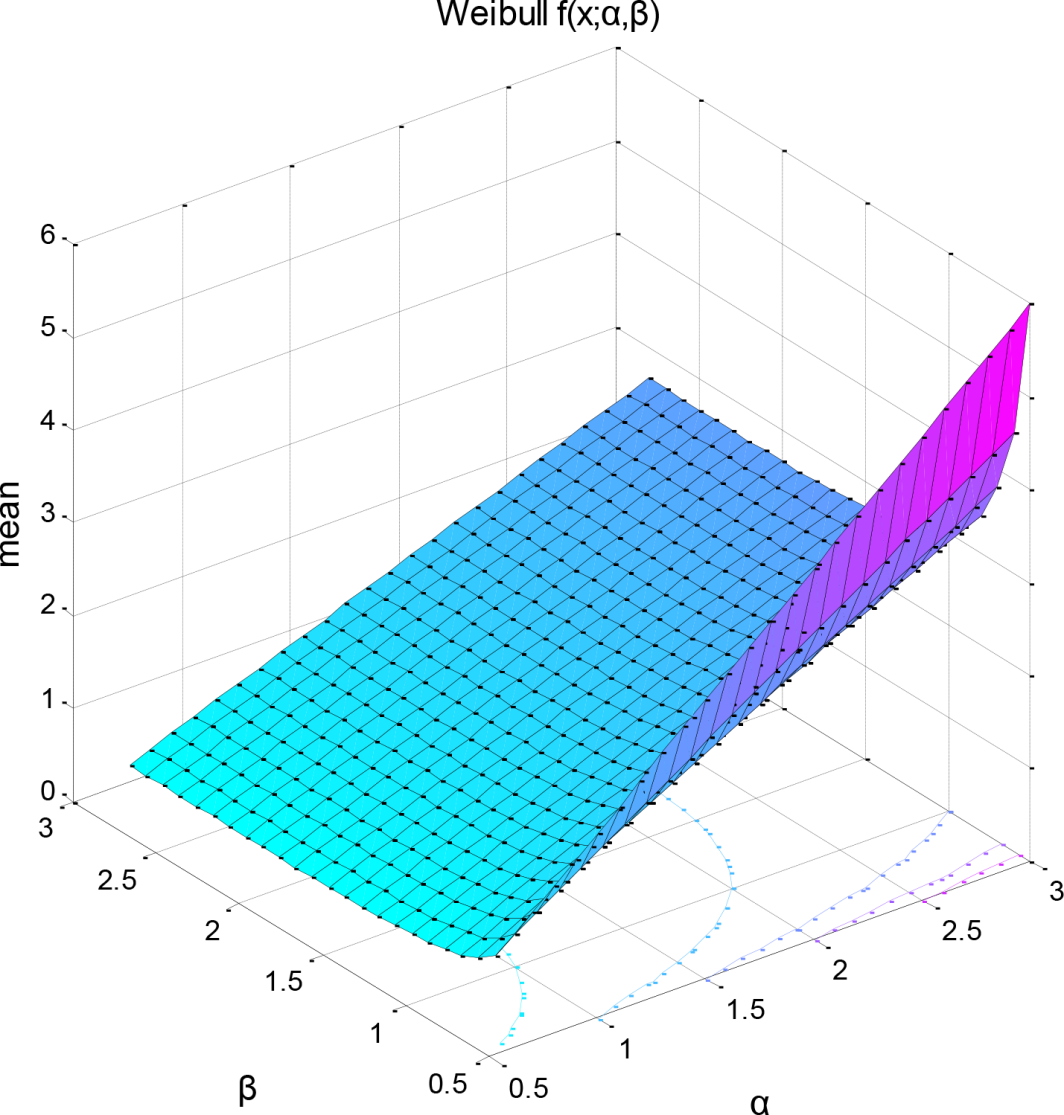
Bivariate 3D interpolation of the mean of the Weibull distribution as a function of its two distribution parameters (mean = 0.6394, var = 0.0417).

**Fig 7:**
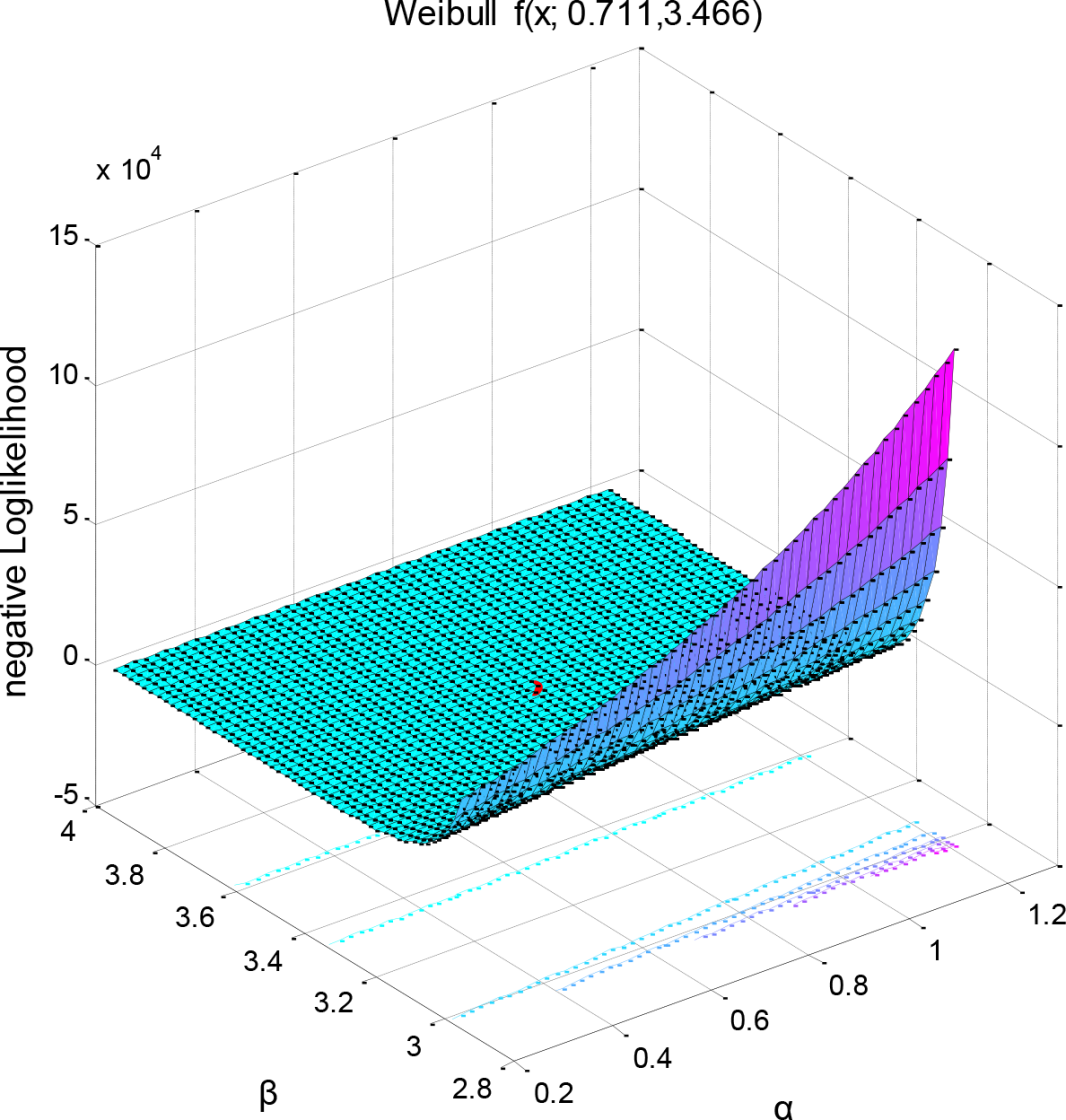
Negative log likelihood surface of a parametric family of distributions (right). The negative log-likelihood function has as input arguments the combination of the two parameter values and was used to return the return the negative of this sum. Here the optimization algorithm to which the values are passed searched for minima rather than maxima. Local minimum is printed in red and was estimated using: $\left(-\text{log}L)=-\text{log}\overset{n}{\underset{i=1}{\prod }}f(x_{i};a,\beta )=-\overset{n}{\underset{\iota =1}{\sum }}\text{log}f(x_{i};a,\beta \right)$.
